# Cold-Induced Urticaria in a Paediatric Patient: A Case Report and Literature Review

**DOI:** 10.7759/cureus.52398

**Published:** 2024-01-16

**Authors:** Madalena Von Hafe, Afonso Caires, Leonor Carneiro-Leão, Diana Bordalo

**Affiliations:** 1 Pediatrics, Centro Hospitalar Universitário de São João, Porto, PRT; 2 Allergy and Immunology, Centro Hospitalar Universitário de São João, Porto, PRT

**Keywords:** temptest, physical urticaria, paediatric allergy, anaphylaxis, cold-urticaria

## Abstract

Acquired cold-induced urticaria is a rare form of physical urticaria, especially in children. The variety of clinical presentations and the low estimated prevalence contribute to its underdiagnosis. Given the associated risk of anaphylaxis, it is crucial to alert clinicians to the different forms of presentation, diagnosis, and treatment. Starting with a case report of acquired cold-induced urticaria in a previously healthy nine-year-old boy, the authors then review the literature about acquired cold-induced urticaria and discuss the diagnostic exams and disease management.

## Introduction

Acquired cold-induced urticaria is a form of physical inducible urticaria expressed by pruritic wheals or angioedema in response to cold exposure [1]. The estimated incidence of cold urticaria is approximately 0.05%, which is considered rare in children [2, 3]. Onset is reported to be most common during the second to fourth decades of life, but presentation at any age is possible [4]. Paediatric studies report a mean age of onset of approximately seven years, which suggests that this disease might be underdiagnosed in the paediatric population [3]. The pathophysiology seems to involve cold-induced de novo formation of auto-antigens and immunoglobulin E (IgE), with subsequent degranulation of mast cells and release of histamine and pro-inflammatory cytokines. Other mechanisms involved include activation of immunoglobulin G (IgG) and immunoglobulin M (IgM) against IgE and mast cells, an example of a type IIb autoimmunity [5]. Studies of the quantification of histamine release show a peak of release within minutes of cold provocation, concomitantly with symptom onset [6].

Here, the authors present a case report of a child with cold urticaria along with a literature review, discuss the limitations of diagnostic tests and disease management, and emphasise the importance of educating patients and their families about the disease course and prognosis.

## Case presentation

A previously healthy nine-year-old boy presented to the outpatient clinic with a history of erythematous and pruritic urticarial wheals in the last five months. These appeared after swimming in the sea and river for about 30 minutes on six occasions. In five of the six episodes, the lesions disappeared in approximately 10 minutes, after skin rewarming, without any other signs or symptoms. On one occasion, gastrointestinal symptoms were reported after swimming in the river. The patient experienced abdominal pain, vomiting, and dizziness, which prompted immediate medical attention. He had no known reactions following contact with water, after exercise, or in association with any food or drug. Interestingly, the child reported previously developing small erythematous and pruritic papules in the exposed areas of the skin after a walk on a cold, windy day.

Given the typical urticarial features of the lesions, a preliminary diagnosis of cold or aquagenic urticaria was made. TempTest® (Courage + Khazaka Electronic, Köln, Germany) was used not only to confirm the diagnosis of cold-induced urticaria but also to establish an initial threshold of 18ºC. The patient was advised to avoid contact with water or cold air below 18ºC. Information on potential symptoms and what to do in the event of another episode or anaphylaxis was provided. The first-line treatment was a daily H1-antihistamine (desloratadine 10 mg) and epinephrine autoinjector (Epipen® 0.15 mg (Meridian Medical Technologies, St. Louis, MO)).

As of the six-month follow-up, there have been no new episodes of urticaria, and a new TempTest® showed a threshold of 11ºC. The patient was instructed to double the antihistamine dose, and at a follow-up evaluation at six months, the temperature threshold decreased again to 8ºC. Eighteen months after the first evaluation, the patient showed complete wheal suppression at 4ºC on the TempTest ®.

## Discussion

Cold urticaria can be classified as typical or atypical [6]. Typical cold-induced urticaria manifests as erythematous, pruritic wheals and may be accompanied by deep tissue involvement with angioedema that occurs within minutes of cold exposure [6]. In these patients, the symptoms are reproduced by cold stimulation testing with an ice cube test [6]. Classically, this involves using a plastic bag containing an ice cube, which is applied to the volar aspect of the patient’s forearm for five minutes and then removed. More recently, TempTest®, a device that can determine the exact temperature that triggers cold-induced urticaria, was developed, as was performed in this case [6]. Although most patients develop localised symptoms, the most feared presentation is cold-induced anaphylaxis, experiencing respiratory or gastrointestinal symptoms, dizziness, hypotension, and shock [7, 8]. Unlike other causes of anaphylaxis, the severity of cold-induced anaphylaxis depends on the exposed area, temperature, and duration of exposure, suggesting a dose-dependent mechanism. This might explain why aquatic activities are the most common trigger for severe systemic reactions. A retrospective study in paediatric patients found a 37% rate of anaphylaxis, with the vast majority of patients experiencing anaphylaxis after aquatic exposure [3].

Additionally, a study by Yee et al. involving paediatric patients with cold-induced urticaria showed that a history of anaphylaxis was more frequent in patients with atopy than in those without atopy [7]. Yee et al. also found that patients with a history of anaphylaxis may experience a negative cold stimulation test (12% negative ice cube test) [7]. Furthermore, the rate of reported systemic reactions is high in patients with cold-induced urticaria, and evidence suggests a higher rate in the paediatric group [9]. This severe presentation might occur in a hospital environment, namely in the operative setting, and the medical community's awareness is imperative [10]. A survey to evaluate the knowledge of this disease among specialists showed that the incidence of systemic reactions in cold-induced urticaria is underestimated, and consequently, epinephrine autoinjectors are underprescribed [11].

Factors influencing clinical presentation are the duration of cold exposure, different temperature thresholds, and the type of contact (cold objects, cold water, cold foods, or cold climate) [12]. Therefore, the type of cold contact might define the clinical presentation; for example, oropharyngeal angioedema is more common after cold food ingestion [12]. It can also be classified into ‘familial’ and ‘acquired’ and further divided into ‘primary’ or 'secondary' depending on underlying causes such as autoimmune and lymphoproliferative diseases, infections, or the use of certain drugs [13]. However, a causal association is often challenging to establish, and its use in daily practice is scarce. Current evidence shows that the mean duration of cold-induced urticaria is approximately six years, with early-onset disease, the presence of atopic comorbidities, and clinical severity being the most established predictors of prolonged duration [12, 13]. However, most studies reporting a disease duration estimate are limited by the follow-up period [3]. A retrospective study showed that in paediatric patients, 78% of children with cold-induced urticaria had a history of atopic disease [7].

The diagnosis of cold-induced urticaria is established based on the clinical history and supported by the cold stimulation test [14]. This test indicates the critical stimulation time threshold, corresponding to the shortest time required to induce a wheel [14]. A study in patients with cold-induced urticaria showed that a critical stimulation time threshold of fewer than three minutes is associated with a higher rate of severe and systemic symptoms [15]. The ice cube test protocol needs to be standardised and may vary between different hospitals, which might explain some of the heterogeneity of the literature results. The H1-antihistaminic drugs and glucocorticoids must be discontinued at least one week before testing to avoid altering the result. The sensitivity of the ice cube stimulating test varies from 53% to 83%, and the specificity from 97% to 10% [16]. The test's sensitivity is lower in paediatric patients than in adults. Evidence shows that almost 50% of paediatric patients with cold-induced urticaria have a negative ice cube test [17]. A prospective National Institute of Health study showed that almost 25% of patients with cold-induced urticaria had a negative cold stimulation test [17]. As such, the recommendation is for epinephrine auto-injectors to be prescribed for all patients with cold-induced urticaria, despite the cold stimulation test result. In fact, many patients with a cold stimulation test lasting more than three minutes or with a negative test may experience systemic reactions [16]. Interestingly, the 2016 European Academy of Allergology and Clinical Immunology (EAACI) Dermatology Section/the Global Allergy and Asthma European Network (GA(2) LEN) task force on urticaria/the European Dermatology Forum (EDF)/Urticaria Network e.V. (UNEV) consensus on the definition, diagnostic testing, and management of chronic inducible urticarias does not mention the use of epinephrine in cold-induced urticaria treatment [14]. In recent years, much effort has been made to understand cold-induced urticaria and its management better. More recently, TempTest® was developed by Charité Medical University in Berlin and is used to define the threshold temperature at which symptoms occur and to document remission. Cold stimulation tests can give inconclusive results, especially when there is an interdependence between cold and other physical triggers [1]. Adapting the cold challenge to real-life conditions may be necessary to induce the patient's symptoms. In addition, diagnosing cold urticaria in children poses challenges due to their limited ability to explain symptoms accurately, potential difficulty distinguishing triggers, the variability in symptoms, and the difficulty that sometimes exists in them cooperating in the cold stimulation test.

Managing cold urticaria includes avoiding triggers such as cold-water immersion (water temperature preferably >25ºC) or rapid systemic cold exposure. Medical therapy consists of prophylactic use of second-generation H1-antihistamines, and the off-label use of other drugs might be helpful in refractory cases [18]. Although standard doses of second-generation H1-antihistamines are often used, up to four times the standard dosage may be required to prevent cold-induced urticaria effectively [18]. The most common off-label drugs used are omalizumab, anakinra, and reslizumab [14, 19, 20]. Omalizumab is an anti-IgE recombinant humanised monoclonal antibody with robust evidence of its efficacy and safety in acquired cold-induced urticaria [20]. As aforementioned, patients should always carry an epinephrine auto-injector, and families should be aware of the symptoms and the risk of anaphylaxis. Trigger thresholds should be measured in all patients, when possible, to monitor treatment efficacy. The literature reports a resolution rate between 5% and 7.5% in the paediatric population, and the severity of presentation might influence the resolution rate [7].

Although the understanding of this pathology has increased dramatically over the last few years, many topics still need to be fully clarified. Disease pathogenesis, typical course, associated comorbidities, predictive biomarkers, diagnosis, and individualised treatment remain unanswered, and even more so when it comes to children. There are few studies addressing this topic in paediatrics, making this diagnosis sometimes difficult for those who frequently deal with children. Furthermore, the lack of literature prevents a well-founded knowledge of the history of the disease in children. Cold urticaria remains a fascinating area of research, and further studies in paediatrics are needed to better characterise the clinical presentation of typical and atypical cold urticaria, correctly establish the prevalence in different age groups, and understand the clinical course and prognosis. What the future holds for us to answer all these questions may be based on genetics, which could also be a promising future in terms of therapy.

## Conclusions

Cold-induced urticaria has become an increasingly recognised pathology in the paediatric age group. This case report aims to highlight the risk of systemic reactions and the importance of preventive strategies. Cold-induced urticaria should be promptly recognised and managed due to its potential life-threatening presentation, and current evidence suggests that an epinephrine auto-injector should be considered in all paediatric patients with suspected cold-induced urticaria, even with a negative cold stimulation test. Most evidence on cold-induced urticaria is drawn from the adult population, with little data available for children. Thus, more studies are needed in this age group to better understand the risk factors, the therapeutic response to different drugs and doses, and the long-term prognosis.
